# Quantifying Information Exchange Between Cells in Inflammaging

**DOI:** 10.3390/bioengineering13020222

**Published:** 2026-02-14

**Authors:** Israr B. M. Ibrahim, Ramana M. Pidaparti

**Affiliations:** 1 Department of Mechanical and Industrial Engineering, Universitas Syiah Kuala, Banda Aceh 23111, Indonesia; 2 College of Engineering, University of Georgia, Athens, GA 30602, USA

**Keywords:** transfer entropy, inflammation, agent-based model, network topology

## Abstract

Inflammaging is inflammation caused by altered cell–cell communications due to aging which leads to impaired wound healing. In this study, we investigated the underlying dynamics of information exchange between macrophage and fibroblast cells during inflammation using an in silico agent-based model. Information exchange was inferred through migration dynamics of motile cells, and network representations of cell–cell information exchange were built. We investigated information exchange through computational modelling during inflammation with two different courses: inflammation followed by full recovery, and inflammation followed by sustained injury due to aging progression. We found that inflammaging leads to reduced cell–cell information exchange and noisier dynamics of cells compared to normal inflammation. Normal inflammation favours higher centrality on the fibroblast nodes, while inflammaging prefers networks with more centrality on the macrophage nodes. This network topology may indicate the primary agents in the inflammation and provide a way to describe inflammation and its course by a network of cell–cell interactions.

## 1. Introduction

Many phenomena in nature emerge as a result of interaction between components. For example, the gradient of concentration in diffusion emerges from interaction between particles, and ant colonies and swarms of birds emerge from interactions between individual animals; the same principle applies to human social structures. Interaction here is defined as an exchange of state or information between individual units/agents.

There are several examples of interactions between agents in natural systems. For example, in the case of particles, collision exchanges momentum between individual particles, and in swarms of birds and fish, individuals exchange information about their position [1]; ants communicate individuals’ positions during bridge formation [2] and use this information, along with an individual’s memory, in foraging [3]; the congregations of photosymbiotic marine flatworm *S. roscoffensis* that form dense biofilms on beaches can be explained by local interactions between individual worms [4]; the dynamic organisation of human crowds relies on local interactions between individuals and their neighbours [5]; information propagation has been used as a basis to explain the collective motion of fish flocks [6]; and individual *E. coli* in a dense suspension synchronise their motion with respect to their neighbours [7]. The collective dynamics of bacteria can change the properties of the fluid [8,9], or, in other words, collective dynamics of individual agents can drive the state of the global system.

Another system that relies on interaction between units/agents is the immune system. It relies heavily on signal transduction between cells [10,11]. Cells have two main methods of communication: by diffusive agents (e.g., cytokines) and by active agents (e.g., cargo). An impairment in cell communication leads to all sorts of disorders and diseases [12,13,14]. Accordingly, it has been proposed that cells naturally form “social networks” [15]. In addition, spatial patterning and self-organisation of cells also emerges from diffusion-based communications [16]. Alteration of cell–cell communication is known to be associated with aging and consequently affect inflammatory response (termed inflammaging) [17]. Recent advances have further elucidated the mechanisms underlying inflammaging, including the senescence-associated secretory phenotype (SASP), altered cytokine networks, and impaired tissue-resident immune cell interactions [18,19,20,21], which consequently change the collective dynamics. Furthermore, it can be reasoned that the course of inflammation might be determined by cell–cell interactions. Hence, analysis of cell–cell interactions may provide insight into the fundamental aspects of inflammation as a process. Analysis of cell–cell communications within the scope of collective behaviours is important since new dynamics appear when cells move as part of a collective [22,23].

Due to its importance, a number of studies have analysed cell–cell communication and its spatial and temporal aspects [22,24,25,26]. Recent technological advances, including single-cell and spatial transcriptomics approaches combined with computational modelling, have provided unprecedented resolution for analysing intercellular interactions during inflammation [27,28]. Experimental platforms such as microfluidic devices with image-tracking have been developed to quantify the spatio-temporal effects of macrophage and fibroblast communication [16]. Even though spatio-temporal effects are important in cell-to-cell communication, the information exchange between cells is also important and needs to be considered to better understand cell functions.

In this study, we investigate collective dynamics in terms of cell–cell information exchange in cases of inflammatory response in normal and aging conditions, and attempt to explain how inflammation time course relates to or emerges from interactions between macrophage and fibroblast cells. We are motivated by our previous study [29], which indicated that the period of healing during inflammation may be explained by migration dynamics and self-organisation of the motile cells. The motile cells (macrophages and fibroblasts) start as random-walking automata. However, once certain conditions are fulfilled and diffusive agents are released, their random-walk is biased toward the location in their neighbourhood with the highest concentration of cytokines (a simplification of chemotaxis). Macrophage walk is biased by TNF-α (tumour necrosis factor-alpha, a pro-inflammatory cytokine, hereafter TNF), and fibroblast walk is biased by TGF-β (transforming growth factor-beta, an anti-inflammatory cytokine, hereafter TGF). We model TNF-α and TGF-β as representative pro- and anti-inflammatory signals; this simplification captures the essential antagonistic feedback loop while omitting other mediators (IL-1, IL-6, IL-10, etc.) for model parsimony. A set of parameters leads to total recovery, while others lead to sustained injuries.

**Figure 1:**
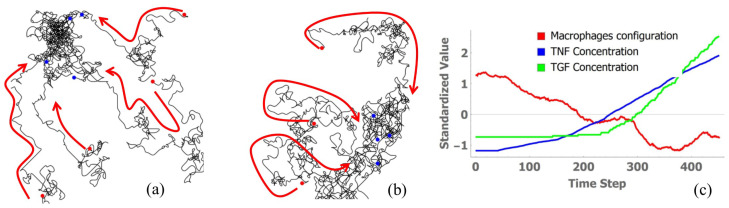
(**a**,**b**) Tracks of macrophages during simulation up to a time step of 500, showing convergence. Red dots indicate starting location of motile cells automata, blue dots indicate location of motile cells after 500 iterations. The arrows indicate the direction of motion of the cells. (**c**) Shannon’s entropy of macrophage configuration is inversely proportional to average concentrations of TNF and TGF on the grid. Time step represents model iterations (dimensionless). All values were standardised using z-score normalisation: z=(x−μ)/σ, where μ is the mean and σ is the standard deviation.

A sample of motile cell migration is shown in Figure 1. Figure 1a,b show the path of some motile cells after 500 iterations. Motile cells initially exhibit random walks, but as shown in Figure 1a,b, the movements of motile cells began converging into particular regions after a certain time. This convergence corresponds to the time the cytokines took to spread over the grid by diffusion. When the configuration of macrophages on the grid is presented as Shannon’s entropy (Figure 1c); it can be seen that the shift in entropy is incidental with the shift in average TNF (pro-inflammatory cytokine) and TGF (anti-inflammatory cytokine) concentrations on the grid. Hence, we can conclude that cytokine secretions and spread alter the migration paths of the motile cells. If we view communication as a process of information exchange that leads to the alteration of observed behaviour between agents/individuals, then these migration path deviations can be viewed as occurring as a result of information exchange between cytokine-secreting motile cells, and this finding can be utilised to infer information exchange between motile cells during inflammation.

These migration dynamics provide an opportunity to infer information exchange between individual macrophages and fibroblasts. We employ concepts from information theory and network science to quantify this information exchange and relate it to the course of inflammation.

## 2. Method

### 2.1. Agent-Based Model of Inflammation

The agent-based model is a grid-based 2D model similar to Cellular Automata. The model consists of four agents: epithelial cells, motile cells (including macrophage and fibroblast), diffusing substance (cytokines), and physical variables representing mechanical stimulation. Each agent has its own states (such as “alive” or 0) and executes rules to evolve these states.

The mechanical stimulation parameter (*S*) represents the magnitude of tissue injury or strain that initiates the inflammatory response. *S* is a dimensionless parameter ranging from 0 (no stimulus) to 1 (maximum stimulus), implemented by biasing the initial activation probability of macrophages. At S=0, no inflammation is triggered and cells exhibit purely random walk behaviour. Higher *S* values proportionally increase the likelihood of initial macrophage activation. The specific values S∈{0,0.2,0.4,0.8} used in this study represent absent, mild, moderate, and severe injury levels, respectively. In biological terms, *S* could represent the magnitude of mechanical strain, pathogen load, or damage-associated molecular patterns (DAMPs) that initiate inflammatory cascades. The 2D approximation is appropriate for modelling epidermal and epithelial inflammation where cells migrate primarily on a substrate layer.

**Figure 2:**
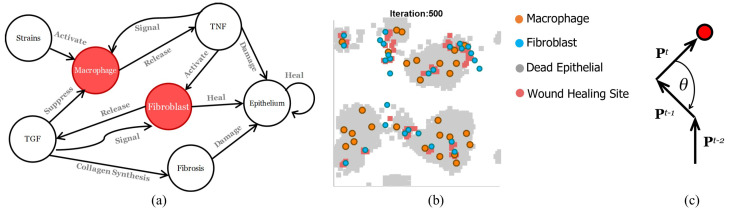
(**a**) Interaction between components in the agent-based model of inflammation [29]. Red circles highlight the agents discussed in this study. (**b**) A representative snapshot of the agent-based model simulation at iteration 500, showing the spatial distribution of macrophages (orange), fibroblasts (blue), dead epithelial cells (grey), and wound healing sites (pink) across the 2D grid. (**c**) A path of migration of a motile cell (red circle). θ is the angle between vectors of the motile cell’s path, *P*. The time step is denoted by the superscript *t*.

Figure 2 illustrates the interactions between agents in the model. The rules are summarised as follows:

1. When macrophages and fibroblasts are activated, they synthesise cytokines. Activation is randomly determined, but biased toward higher values of cytokines present in the cell’s neighbourhood.
2. Macrophages are activated by the presence of stimuli (*S*), and release pro-inflammatory cytokines (TNF). TNF synthesis is probabilistic according to the level of TGF. Fibroblasts are activated by the presence of pro-inflammatory cytokines, and release anti-inflammatory cytokines (TGF). TGF synthesis is probabilistic according to the level of TNF.
3. Both macrophages and fibroblasts exhibit random walks. Macrophages and fibroblasts are signalled by different cytokines [30]; specifically, macrophages are signalled by TNF-alpha (pro-inflammatory) and fibroblasts by TGF (anti-inflammatory). Macrophages’ random walk is biased toward higher concentrations of TNF. Fibroblasts’ random walk is biased toward higher concentrations of TGF.
4. Epithelial cells change their state to death (apoptosis) in the presence of TNF in their neighbourhood and change their state to alive in the presence of TGF; the latter is called healing. When the healing condition is triggered, the neighbourhood will be randomly assigned at least one fibrosis site, which will create further risk of injury to epithelial cells in the neighbourhood (change the state of epithelial cells to death). Apoptosis depends on the level of TNF [31].
5. Cytokines diffuse according to discrete forms of the diffusion equation,
  (1) $$d\phi /dt=D.\nabla^{2}\phi -K.\phi$$
  where ϕ is the cytokine’s concentration, *D* is the diffusion constant that controls the spread rate of the cytokine, and *K* is the degradation constant that controls the decay rate of the cytokine, accounting for cytokine clearance and proteolysis. The −Kϕ term represents first-order degradation kinetics. Typically, D>K.
  For quick reference and to enhance reproducibility, Table 1 provides a summary of these rules in tabular form. The detailed mathematical formulations are provided in the enumerated list above.

Figure 2b shows a sample of simulation results with the distribution of wounds (dead epithelial cells) and motile cells (macrophage and fibroblast). When stimuli are not present, i.e., *S* = 0, the migration dynamics of motile cells are dominated by random walks (see Video S1 in Supplementary Materials). When stimuli are present, cytokines are released, and migration dynamics change to biased random walks (see Video S2 in Supplementary Materials).

### 2.2. Aging Conditions

The basis of our aging progression modelling is delayed healing, a hallmark of inflammaging [32,33]. Aging is characterised by increased levels of pro-inflammatory cytokines [34], impairment of wound repair and tissue regeneration [33], and prolonged fibrosis [35,36]. We implemented three aging conditions—Normal (*N*), Aging 1 (A1), and Aging 2 (A2)—by adjusting four key model parameters that reflect these biological hallmarks. Table 2 summarises the parameter values for each condition.

These parameter adjustments reflect known physiological changes: (i) enhanced pro-inflammatory cytokine production (TNF synthesis encouraged), (ii) increased sensitivity to apoptotic signals, (iii) impaired tissue regeneration capacity, and (iv) prolonged fibrotic response. The progression from Normal to Aging 2 represents increasingly severe aging-related dysfunction, yielding correspondingly more severe sustained injuries during inflammation.

### 2.3. Transfer Entropy and Information Exchange

Concepts from information theory and statistical mechanics have been used to provide analyses of biological systems, such as quantifying information flow and spatial dynamics in ecological populations by employing an extended form of mutual information [37] and analysing social dynamics in animal groups [38].

A measure of information exchange is transfer entropy. This concept has been applied to explain self-organisation in cellular automata [39], to determine leader–follower interactions in zebra fish flocks [40], to measure interaction between left and right lungs to infer disease [41], and to infer causal interaction in electroencephalography data [42]. More recently, information-theoretic approaches have been extended to analyse biochemical signalling pathways, collective cell behaviour, and multicellular coordination [43,44].

Transfer entropy is an extension of mutual information, which is a measure of common information shared between two random variables. Mutual information between random variables *X* and *Y* is expressed as

(2) I(X;Y)=H(X)+H(Y)−H(X,Y)

where H(X) is the information entropy of *X* and H(X,Y) is the joint information entropy of *X* and *Y*. Expanding the concept of mutual information, one can define conditional mutual information, which is a measure of shared information between *X* and *Y*, given another variable *Z*,

(3) I(X,Y|Z)=H(X|Z)+H(Y|Z)−H(Z,Y|Z)

In this study, we aim to quantify shared information between macrophages and fibroblasts given their migration dynamics during inflammation. The direction of migration can be represented as an angle between current and previous paths taken by a cell, i.e., $\theta_{t,t-1}=\cos^{-1}\frac{P^{t}.P^{t-1}}{|P^{t}\left|\right|P^{t-1}|}$, where $P^{t}$ is the vector of a path of a cell at time *t* (Figure 2b). We denote this angle by $M_{t}$ and $F_{t}$ for macrophages and fibroblasts, respectively. Transfer entropy, $T_{F\to M}$, between $M_{t}$ and $F_{t}$ can be defined as mutual information between the two random processes conditioned on a previous instance of $M_{t}$. It is expressed as

(4) $$\begin{array}{cc} T_{M\to F} & =I(F_{t-1};M_{t}|M_{t-1})\\ = & \sum_{M_{t}\in X_{t}}\sum_{M_{t-1}\in X_{t-1}}\sum_{F_{t-1}\in Y_{t-1}}p\left(M_{t},M_{t-1},F_{t-1}\right)\times \log\frac{p(M_{t}|M_{t-1},F_{t-1})}{p(M_{t}|M_{t-1})} \end{array}$$

where $p(M_{t}|M_{t-1},F_{t-1})$ is the probability of $M_{t}$ given $M_{t-1}$ and $F_{t-1}$. The probabilities $p(M_{t},M_{t-1},F_{t-1})$ are calculated by generating joint probability densities using histogram binning. Migration angles θ∈[0,2π] were discretised into 20 bins, yielding a bin width of Δθ=π/10 (approximately 0.314 radians). This binning resolution was chosen to balance statistical power (sufficient samples per bin from 10 simulation replicates) with information resolution. Sensitivity analysis showed that transfer entropy estimates were robust across bin counts ranging from 15 to 25 bins.

Transfer entropy in this case can be thought of as the reduction in uncertainty of predicting the direction of a fibroblast at time step *t* given its previous direction (at t−1) and the direction of a macrophage. Since macrophages release TNF, which triggers fibroblasts to signal each other (see Figure 2a), and in return, fibroblasts release TGF, which suppresses signalling between macrophages, transfer entropy $T_{M\to F}$ can therefore be seen as an estimation of the degree of influence a macrophage has on each fibroblast.

## 3. Results

**Figure 3:**
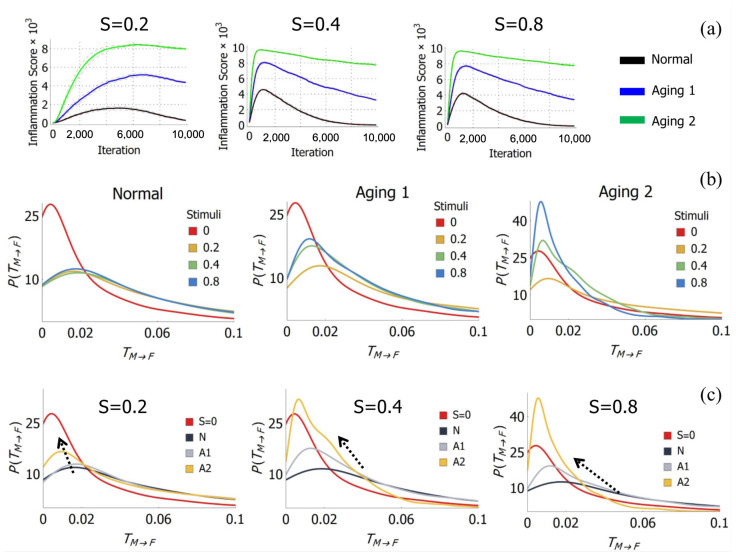
(**a**) Inflammation time course under three aging progression and stimuli levels. Inflammation score (y-axis) represents the number of dead epithelial cells, or injured cells. Inflammation score is the average (mean) of ten repeated simulations; red dashed line indicates standard error. Inflammation course in Normal cases (dark) ended with full recovery, while Aging 1 (blue) and Aging 2 (green) cases carried sustained injuries of different severity. Aging 2 caused more severe sustained injury than Aging 1. (**b**,**c**) Distribution of $T_{M\to F}$ (estimated PDF) under (**b**) stimuli (S=0.2, S=0.4, S=0.8) and (**c**) aging progression (Normal (*N*), Aging 1 (A1) and Aging 2 (A2)). Notice the distribution shifts toward S=0 (red line) as aging progresses from *N* to A1 and eventually A2. The arrow indicates direction of shift.

We carried out agent-based simulations and extracted the tracks of macrophages and fibroblasts at each time step. The simulations were carried out under four levels of mechanical stimuli, *S*: 0, 0.2, 0.4 and 0.8, and three aging conditions, termed Normal (*N*), Aging 1 (A1) and Aging 2 (A2), with 20 macrophages and 20 fibroblasts. Figure 3a shows the inflammation time course for all aging cases and stimuli levels. The zero stimuli S=0 cases do not provoke any response, so the inflammation score is flat, and no cell signalling cytokines are released.

As described in Methods (Section 2.2), the three aging conditions represent progressively severe aging-related dysfunction. Simulation results show that these parameter sets yield increasingly severe sustained injuries (inflammation scores) during the simulation time course, as is evident in Figure 3a. Thus, we refer to the aging progression order as N>A1>A2.

### 3.1. Transfer Entropy

We repeated simulations of each stimuli level and aging progression ten times. Figure 3b,c show the estimated distribution of $T_{M\to F}$ for each stimuli level and aging progression across all ten repeated simulations.

As can be seen in Figure 3b,c, the no-stimuli (S=0, red lines) cases lead to a high likelihood of low $T_{M\to F}$ (close to zero). It is apparent that aging progression leads to a shift in $T_{M\to F}$ distribution toward the $T_{M\to F}$ distribution of no-stimuli cases (S=0). Thus, the migration dynamics of cells becomes progressively noisier (less coordinated, characterised by lower transfer entropy values) as aging progresses, with cell movements approaching random-walk behaviour. This indicates weak information exchange between macrophages and fibroblasts under aging conditions.

### 3.2. Transfer Entropy ($T_{M\to F}$) Networks

**Figure 4:**
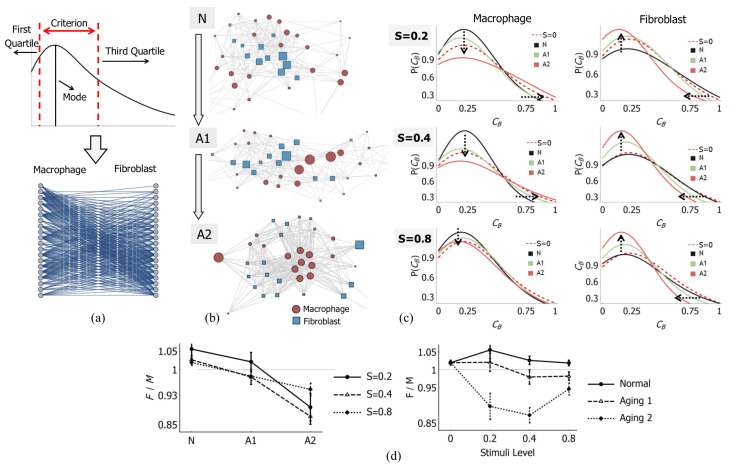
(**a**) Top: A range of $T_{M\to F}$ values (criterion) used for edge generation. The image illustrates a typical $T_{M\to F}$ distribution (as in Figure 3a), along with its properties. Bottom: A sample bipartite graph showing macrophage and fibroblast nodes connected together. (**b**) A sample of networks generated for Normal (*N*), Aging 1 (A1) and Aging 2 (A2) cases with S=0.4. Centrality is visualised by the size of the nodes. The red disc denotes macrophage nodes; the blue square denotes fibroblast nodes. Top (*N*): a network which prefers low centrality nodes on the macrophage side. Middle (A1): a network which has no preference. Bottom (A2): a network which prefers low centrality on the fibroblast side. Each is associated with an inflammation course of Normal, Aging 1, and Aging 2 conditions (Figure 3a). (**c**) Betweenness centrality distribution for each case. Dashed line indicates S=0 cases. Arrows indicate shift in likelihood of nodes’ centrality by aging progression (Normal, *N* to Aging 1, A1 to Aging 2, A2). (**d**) Ratio of fibroblasts’ (F) and macrophages’ (M) betweenness centrality (average of 10 simulation trials). Vertical lines at each data point denote standard error.

To investigate information exchange further, we constructed a network connecting a macrophage to a fibroblast, which is presented in a bipartite graph (Figure 4a). $T_{M\to F}$ values were used as the basis for generating the edges of the graph. Based on the results of the $T_{M\to F}$ distribution, we took the most likely $T_{M\to F}$ values to determine the edge generation criteria (Figure 4a). We defined a range of values with the mode of distribution as a point of reference. We decided to use the first and third quartiles of each distribution as the bounds of the criterion and to account for asymmetry of the distribution. Figure 4a illustrates the criterion used as a basis for edge generation.

The edge generation procedure is as follows: for each estimated $T_{M\to F}$ between a pair of macrophages and fibroblasts (per stimuli level and aging progression), if said pair had $T_{M\to F}$ within the range, an edge was generated. Since the networks are bipartite, we may resolve the network into macrophage and fibroblast parts. Figure 4c shows the estimated betweenness centrality distribution of macrophage–fibroblast $T_{M\to F}$ networks for all ten simulation trials. Figure 4b visualises samples of these networks.

The most prominent feature in Figure 4c is the opposite preference between macrophage and fibroblast nodes due to aging. As aging advances (from *N* (dark) to A1 (green) to A2 (red)), the likelihood of high-centrality nodes on the macrophage side is raised, while the fibroblast side prefers low-centrality nodes (the shifts are illustrated by arrows in Figure 4c). This conflicting relationship applies at all stimuli levels (as seen in Figure 4c). In addition, the S=0 cases show less preference of centrality (Figure 4c, dashed line).

We also calculated the sum of the centrality of macrophage and fibroblast nodes (denoted by *F* and *M*, respectively) and averaged them over all ten simulation trials for each case. Figure 4d shows the ratio, F/M. As can be seen in Figure 4d, F/M fell below 1.00 with aging conditions, indicating the shift in centrality from the fibroblast nodes to the macrophage nodes. In terms of stimuli level, although the shift in distribution is slight, a preference of centrality can be observed in terms of F/M. As seen in Figure 4d, the networks for aging cases lead to F/M<1.00 in general, indicating that centrality is more likely on the macrophage nodes. In normal cases, F/M is always bigger than 1.00, indicating that centrality is more likely on fibroblast nodes. Hence, we have a divergent trend in terms of stimuli level.

## 4. Discussion

### 4.1. Characteristics of Information Exchange Under Different Stimuli and Aging Progression

Figure 3b,c indicate that in the presence of cell signalling cytokines (when stimuli S>0), transfer entropy increased. However, the skewness type of the distributions is the same, and there is only a slight difference between transfer entropy distributions from cases at different stimuli levels (Figure 3b,c, S>0). On the other hand, aging progression reduced transfer entropy and shifted transfer entropy distribution toward S=0 cases. This shift indicates that the collective dynamics of motile cells in aging cases become noisier—characterised by reduced transfer entropy and less predictable migration patterns—approaching the random-walk behaviour of S=0 cases, where no cytokine-mediated coordination occurs.

However, Figure 3b,c only capture the landscape of transfer entropy and coarse-grain the individual relations between macrophage and fibroblast cells. The network representation of the information exchange helps to unravel the relation between individual macrophage and fibroblast cells. It is revealed that aging conditions lowered the likelihood of low-centrality nodes in the macrophage part, while raising the likelihood of centrality in the fibroblast part (Figure 4c). For S=0 cases, there seems to be less preference.

Based on observations of the $T_{M\to F}$ distributions (Figure 3b,c) and its network properties (Figure 4c), we are able to distinguish collective dynamics underlying different cases of stimuli and aging conditions. The no-stimuli (S=0) cases have less preference of centrality (Figure 4c) and low $T_{M\to F}$ values (Figure 3b,c). We may interpret this as a signature of collective cell dynamics driven by environmental factors without any diffusion-based signalling medium, since there was no cytokine involved.

The Normal (*N*) cases had higher likelihood of high $T_{M\to F}$ and centrality the of $T_{M\to F}$ network in the fibroblast part, which may be indicative of collective cell dynamics driven strongly by diffusion-based signalling. Aging 2 (A2) cases had more likelihood of low $T_{M\to F}$, similarly to the no-cytokine cases, but the $T_{M\to F}$ networks preferred centrality in the macrophage part. Since TNF secretion was strongly encouraged, collective dynamics in Aging 2 cases are assumed to be driven by diffusion-based signalling mediums, which may have encouraged interactions centred around the macrophage.

Lastly, in Aging 1 cases, the $T_{M\to F}$ values are rather high (Figure 3b,c), but the $T_{M\to F}$ networks have less preference for centrality, similarly to no-stimuli cases (Figure 4c,d). Thus, collective dynamics in Aging 1 cases are distinct from other cases, and may be more similar to Normal and no-cytokine cases than Aging 2 cases.

We conclude that each condition (aging and stimuli level) displays distinct collective dynamics and may be characterised by $T_{M\to F}$ and its network. Specifically, collective cell dynamics driven by diffusion-based signalling can be distinguished from ones without diffusion-based signalling.

### 4.2. Relation Between the Course of Inflammation and Macrophage–Fibroblast $T_{M\to F}$ Network

In Figure 3a, it can be observed that the inflammation time course in Normal cases eventually ends with full recovery, while Aging 1 and Aging 2 conditions lead to sustained injuries of different intensity. Based on Figure 3a and Figure 4c, it is possible to associate node centrality preference with the course of inflammation.

All inflammation followed by full recovery (i.e., all Normal cases, black lines in Figure 3a) appears to prefer $T_{M\to F}$ networks with lower centrality in the macrophage nodes (black lines in Figure 4c), shifting the centrality toward fibroblast nodes. A sample of these networks is presented in Figure 4b, marked by *N*. Inflammation that leads to sustained injury (i.e., aging cases, blue and green lines in Figure 3) tends to have $T_{M\to F}$ networks with opposite preference: higher centrality in the macrophage nodes. Samples for the latter case are visualised in Figure 4b, marked by A1 and A2, respectively. Therefore, the course of inflammation (i.e., full recovery or sustained injury) can be associated with particular $T_{M\to F}$ networks.

The $T_{M\to F}$ networks presented here may have encoded the primary role of the agents in the system, or the degree to which agents drive the system. Fibroblasts are assigned as the agent that administers healing and suppresses inflammatory cytokines. Hence, the preference of centrality in the fibroblast nodes in full-recovery cases might be indicative of its role. It might also indicate that fibroblasts were the primary driver of the system for those cases. Similarly, macrophages are assigned as the agent that exacerbates inflammatory response and is the primary motivator of injury. Preference of high-centrality nodes in the macrophage side may be indicative of its role or influence in the system.

Another interpretation of the results is that macrophage–fibroblast $T_{M\to F}$ networks may govern the course of inflammation (i.e., full or partial recovery). Consequently, the course of inflammation may be modified by altering macrophage–fibroblast $T_{M\to F}$ networks. Th information exchange estimated in this study is not solely driven by the secretion and diffusion of cytokines, but also environmental factors such as motile cell velocity and local density. A motile cell avoids collision with another motile cell, and a crowded area can deter the movement of motile cells. In real-life circumstances, biological fibres are also known to deter motile cells migration, impairing immune response [45]. Thus, information exchange between macrophages and fibroblasts can be altered by modifying mechanical and environmental factors. Some authors have reported methods to alter cell–cell interactions through mechanical means [46,47].

The agent-based model in this study only includes two general types of cells (macrophages and fibroblasts) and cytokines to represent pro- and anti-inflammatory mediators. However, it is able to capture events found commonly found during inflammation, as described in various studies [48,49,50]. Furthermore, the data used in this study to infer information exchange does not require knowledge of the inner aspects of the in silico model. The data (i.e., migration path) is based on the observable aspects of the model. This data can be substituted by imaging-based motion-tracking data. This approach can be used to quantify relations between other type of cells and their interactions.

### 4.3. Model Validation and Comparison to Experimental Observations

Our simulation results exhibit inflammation time courses consistent with experimental observations. The Normal condition simulations (Figure 3a, black lines) show the characteristic trajectory of acute inflammation: a rapid rise in inflammatory markers (injury score) within initial time steps, reaching a peak, followed by resolution and full recovery. This pattern aligns with well-documented inflammation dynamics in healthy tissue repair. The sustained injury observed in Aging conditions (*A*1 and *A*2) corresponds to delayed wound healing and chronic inflammation reported in aged animal models and clinical studies. Specifically, recent experimental studies demonstrate delayed wound closure (7–14+ days in aged vs. 3–7 days in young subjects), prolonged macrophage infiltration, and impaired M1-to-M2 transition in aged wounds [51,52,53], consistent with our *A*1 and *A*2 simulation results showing sustained inflammation scores.

#### 4.3.1. Cell Migration Dynamics and Intravital Imaging Data

Our model’s migration dynamics can be compared with quantitative data from intravital imaging studies. Intravital multiphoton microscopy of wound healing reveals that macrophages migrate more slowly than neutrophils, with recruitment peaking at 24–48 h post-injury [54,55], consistent with our model’s chemotactic response patterns. Fibroblasts exhibit collective, swarming-like migration driven by N-cadherin upregulation, with measurable velocity and directionality during scar formation [56]. Our simplified chemotaxis rules (biased random walk toward cytokine gradients) qualitatively capture these migration patterns, though they do not explicitly model the molecular determinants of directionality such as integrin–ECM interactions or cadherin-mediated cell–cell adhesion.

Importantly, experimental studies demonstrate that chemotactic indices (measures of directed migration) are substantially reduced in aged cells. Macrophages from older adults show impaired migration toward chemotactic signals with reduced cytoskeletal rearrangement [57], while aged fibroblasts lose responsiveness to chemotactic cues including TGF-β and hypoxia [58]. Our aging parameter adjustments (reduced epithelial regeneration, prolonged fibrosis, enhanced TNF synthesis) indirectly capture these effects by altering the cytokine landscape and healing dynamics, though future models could explicitly incorporate age-dependent migration velocities and gradient-sensing capabilities, as demonstrated experimentally [59,60,61].

#### 4.3.2. Validation Approaches from Computational Modelling Studies

Computational models of macrophage–fibroblast interactions employ multiple validation strategies that inform the interpretation of our results. Parameter estimation approaches include calibration to transcriptomic data from in vitro co-cultures matched to ex vivo tissue profiles [62], quantitative comparison of simulated cell distributions with immunofluorescent images using sensitivity analysis [63], and direct parameter extraction from cell-tracking experiments [64,65]. Model predictions are validated by comparing inflammation time courses, cytokine profiles, and tissue-level outcomes with experimental data, with quantitative agreement typically ranging from 9 to 19% error for well-calibrated models [63,65].

Our model employs a similar validation philosophy: the simplified rule set generates emergent dynamics (inflammation time courses, network topologies) that are compared qualitatively with known biological patterns rather than fitted to specific datasets. This approach prioritises mechanistic insight over parameter precision, trading quantitative accuracy for interpretability. The transfer entropy framework provides a novel lens for analysing information flow that complements traditional validation metrics focused on cell counts and cytokine concentrations.

#### 4.3.3. Biological Interpretation of Network Centrality

The antagonistic relationship between macrophage (pro-inflammatory) and fibroblast (pro-healing) network centrality observed in our model aligns with recent experimental findings. Studies show that balanced macrophage–fibroblast communication, particularly through TNF-α and TGF-β signalling and spatial co-localisation, is critical for proper healing outcomes [62,66]. Dysregulation of this balance—such as sustained M1 macrophage dominance or impaired fibroblast activation—leads to chronic inflammation or pathological fibrosis, consistent with the network topology shifts we observe in aging conditions.

Resolution of inflammation is marked by specific molecular transitions including the switch from pro-inflammatory eicosanoids to specialised pro-resolving mediators (resolvins, protectins, maresins), M1-to-M2 macrophage phenotype switching with increased IL-10 and TGF-β expression, and enhanced efferocytosis [67,68,69,70]. Our model’s shift in network centrality from macrophages to fibroblasts during successful resolution may reflect these underlying molecular transitions, as fibroblast-dominant networks correspond to TGF-β-mediated healing and macrophage-dominant networks correspond to sustained TNF-α signalling. While our model does not explicitly represent these molecular markers, the emergent network topology may serve as a systems-level readout of resolution state.

Future work should incorporate quantitative validation by comparing predicted transfer entropy values and network topologies with measurements from microfluidic co-culture systems or in vivo imaging studies that track individual cell migration dynamics, enabling direct parameter calibration to experimental migration velocities and chemotactic indices [71].

### 4.4. Model Limitations and Future Directions

Our model maintains constant cell populations (20 macrophages, 20 fibroblasts), whereas physiological inflammation involves significant macrophage recruitment and fibroblast proliferation. This simplification was adopted to isolate the effects of cell–cell information exchange from population dynamics. The observed differences in transfer entropy and network topology therefore reflect changes in communication efficiency rather than cell abundance.

Additionally, our model implements binary activation states (secreting/not secreting) with constant secretion rates. While activation thresholds are concentration-dependent, dose-dependent secretion kinetics observed in real cells are simplified. This approximation captures essential dynamics while maintaining model parsimony but could be refined in future iterations.

We use identical diffusion constants (D) for TNF-α and TGF-β as a first approximation. While these cytokines have different molecular weights (TNF-α 51 kDa as trimer, TGF-β 25 kDa dimer), their diffusion coefficients in tissue are within the same order of magnitude. Given other model simplifications, this assumption is justified, though differential diffusion rates could be explored in sensitivity analyses.

#### 4.4.1. Biological Complexity Not Captured by Current Model

Several age-related biological mechanisms are not explicitly modelled but could inform future extensions. Regarding the Senescent cell accumulation, Aging is characterised by the accumulation of senescent cells secreting the SASP, which includes pro-inflammatory mediators (IL-6, IL-8, TNF-α, PDGF-AA, IL-33) that impair macrophage phagocytosis and fibroblast function [72,73,74,75]. When senescent cells exceed approximately 15% of the wound population, healing is significantly impaired [73]. Our aging parameters indirectly capture elevated pro-inflammatory signalling but do not explicitly model senescent cell dynamics or SASP heterogeneity.

Our model uses uniform migration rules for all age conditions, whereas experimental data demonstrate quantitative reductions in cell motility with age. Aged macrophages show substantially reduced chemotaxis (predicting 70% of lifespan variance) [57,61], while aged fibroblasts exhibit only 37% migration increase with TGF-β compared to 109% in young cells [58]. Aged fibroblasts also show a disorganised actin cytoskeleton and impaired integrin function despite similar integrin expression [59]. Future models could incorporate age-dependent migration velocities, reduced gradient sensing, and impaired ECM navigation to more directly represent these cellular changes.

Our 2D grid does not represent ECM stiffness gradients or fibrotic tissue architecture. ECM stiffness modulates macrophage polarisation via mechanosensitive signalling pathways [76] and influences fibroblast migration and activation [60,77]. Age-related changes in ECM composition and stiffness are important determinants of cell behaviour that merit inclusion in future spatially explicit models.

Our model simplifies inflammation to TNF-α/TGF-β antagonism. In reality, resolution involves coordinated transitions in lipid mediator profiles (from pro-inflammatory eicosanoids to specialised pro-resolving mediators including resolvins and maresins), macrophage phenotype switching (M1 to M2 with specific surface markers like CD83, CD200R), and restoration of homeostatic gene expression [67,68,69,70]. Incorporating these molecular details would enable in silico testing of therapeutic interventions targeting specific resolution pathways.

#### 4.4.2. Recommended Future Extensions

Future extensions should incorporate (1) monocyte recruitment from blood (proportional to TNF levels) and senescent cell dynamics with SASP secretion; (2) fibroblast proliferation (TGF-dependent) and age-dependent impairment; (3) cell apoptosis and egress; (4) age-dependent migration velocities and chemotactic response reduction based on experimental measurements [57,58,59]; (5) ECM stiffness fields and mechanotransduction; and (6) explicit representation of resolution markers to define quantitative recovery criteria.

Validation against experimental datasets should include (a) comparison of simulated migration velocities with intravital imaging measurements [54,56,71]; (b) calibration of age-dependent parameters to measured chemotactic indices and migration responses [57,58]; (c) matching simulated inflammation time courses to experimental wound healing kinetics in young versus aged animal models; and (d) benchmarking network topology predictions against spatial transcriptomic data from inflamed tissues. These additions would enable investigation of how population dynamics, variable cell motility, and molecular resolution pathways interact with information transfer to determine inflammation outcomes.

## 5. Conclusions

Inflammation relies on communication between cells. In this study, we simulated the dynamics of cells during inflammation. We quantified the information exchange between cells to explain the inflammation time course under different conditions, including aging. We found that aging leads to lower cell–cell information exchange and migration of cells is noisier, presumably due to diluted signalling. We also characterised the course of inflammation by constructing networks of cell–cell information exchange and found that normal inflammation courses tend to have network centrality on the fibroblast nodes, while inflammaging cases tend to have network centrality on the macrophage nodes. These centralities may also denote the primary agents of the inflammation process. The approach presented can be extended to other cell types in inflammation processes related to certain diseases.

## Figures and Tables

**Table 1:** Summary of agent-based model rules.

Rule	Condition	Action/Outcome
i. Cell Activation		
Macrophage activation	- Mechanical stimulus present (S>0) - Probabilistic, biased by local TNF concentration	- Activated macrophage: State → 2 - Begins TNF synthesis - Remains mobile
Fibroblast activation	- Pro-inflammatory cytokine (TNF) present - Probabilistic, biased by local TGF concentration	- Activated fibroblast: State → 1 - Begins TGF synthesis - Ceases migration (settles at injury site) - Lifespan reduced by 75%
ii. Cytokine Synthesis		
TNF release (Macrophages)	- Macrophages activated (State = 2) - Probabilistic: $rand\left(\alpha_{3},\beta_{3}\right)\geq {\left[TGF\right]}_{local}$ - Inhibited by TGF	- TNF concentration +1 at cell location - Activator–inhibitor dynamics established
TGF release (Fibroblasts)	- Fibroblasts activated (State = 1) - Probabilistic: $rand\left(\alpha_{4},\beta_{4}\right)\leq {\left[TNF\right]}_{local}$ - Promoted by TNF	- TGF concentration +1 at cell location - Antagonistic feedback to macrophages
iii. Cell Migration (Chemotaxis)		
Macrophage movement	- Macrophages present (activated or not) - Moore neighbourhood sampled	- Biased random walk toward higher [TNF] - Velocity: three cells/iteration - Avoids collision with other cells
Fibroblast movement	- Fibroblasts present (before activation) - Moore neighbourhood sampled	- Biased random walk toward higher [TGF] - Velocity: three cells/iteration - Immobilised upon activation
iv. Epithelial Cell Dynamics		
Apoptosis	- TNF present in neighbourhood - Concentration exceeds apoptosis threshold	- Epithelial cell: State → 0 (dead) - Injury score increases
Healing	- TGF present in neighbourhood - Healing time ($t_{h}$) elapsed	- Epithelial cell: State → 1 (alive) - May introduce fibrosis site randomly - Injury score decreases
v. Cytokine Diffusion and Degradation		
Spatial spread and decay	- Continuous for all cytokines - Reaction–diffusion dynamics	- $\frac{d\phi }{dt}=D\nabla^{2}\phi -K\phi$ - D: diffusion constant (spread rate) - K: degradation constant (decay rate) - Creates concentration gradients

Notes: Notation: rand(α,β) samples from Beta(α, β) distribution; ${\left[TNF\right]}_{local}$ and ${\left[TGF\right]}_{local}$ denote local cytokine concentrations; Moore neighbourhood includes eight adjacent cells. All rules execute asynchronously in each iteration. Detailed mathematical formulations are provided in the enumerated list (Section 2.1).

**Table 2:** Model parameters for aging conditions (with absolute values).

Parameter	Normal (*N*)	Aging 1 (*A*1)	Aging 2 (*A*2)
Cytokine Synthesis			
TNF synthesis probability ($\alpha_{3},\beta_{3}$) *	(1.0, 3.0)	(1.2, 2.5)	(1.5, 2.0)
TGF synthesis probability ($\alpha_{4},\beta_{4}$) *	(2.0, 1.0)	(2.0, 1.0)	(2.0, 1.0)
Epithelial Cell Dynamics			
Apoptosis threshold (TNF level)	0.8	0.6	0.4
Mitosis probability ($P_{mt}$)	0.20	0.16	0.10
Healing time ($t_{h}$, iterations)	5	6	8
Fibrotic Response			
Fibrosis duration ($K_{c}$, iterations)	50	75	100
Collagen damage probability	$N_{c}$/9	$N_{c}$/9	$N_{c}$/9
Diffusion Parameters (constant across conditions)			
TNF diffusion constant ($D_{TNF}$)	0.07		
TGF diffusion constant ($D_{TGF}$)	0.10		
TNF degradation constant ($K_{TNF}$)	$1\times 10^{-3}$		
TGF degradation constant ($K_{TGF}$)	$1\times 10^{-5}$		
Motile Cell Parameters (constant across conditions)			
Macrophage population	20		
Fibroblast population	20		
Cell lifetime ($t_{im}$, iterations)	20		
Cell velocity (cells/iteration)	3		
Repopulation interval ($\epsilon_{pop}$)	5 iterations		

* Beta function parameters used for probabilistic cytokine release: rand(α,β) samples from Beta(α,β) distribution. Higher α/β ratio increases synthesis probability. Aging progression is implemented through (i) enhanced TNF synthesis (increased $\alpha_{3}$, decreased $\beta_{3}$); (ii) increased apoptosis sensitivity (lower threshold); (iii) impaired tissue regeneration (reduced $P_{mt}$ and increased $t_{h}$); and (iv) prolonged fibrotic response (increased $K_{c}$).

## Data Availability

Data is available through this repository: https://doi.org/10.6084/m9.figshare.7283510.

## Supplementary Materials

The following supporting information can be downloaded at: https://www.mdpi.com/article/10.3390/bioengineering13020222/s1, Video S1: A sample of macrophage (red circle) and fibroblast (blue square) migration from a simulation with no stimuli present (*S* = 0) for 500 simulation time steps. No injury (gray square) was present because there were no cytokines released. Motile cell motions are largely random walks. Interaction still takes place as each agent avoids collision with the others; Video S2: A sample of macrophage (red circle) and fibroblast (blue square) migration from a simulation of the Normal (N) case with stimuli *S* = 0.4 for 500 simulation time steps. Cytokines were released by both macrophage and fibroblast cells, strongly steering motile cell motions and driving them to organize in certain regions.
